# Protein expression in experimental malignant glioma varies over time and is altered by radiotherapy treatment

**DOI:** 10.1038/sj.bjc.6603269

**Published:** 2006-09-12

**Authors:** C Wibom, F Pettersson, M Sjostrom, R Henriksson, M Johansson, A T Bergenheim

**Correction to:** *British Journal of Cancer* (2006) **94**, 1853–1863. doi:10.1038/6603190

Owing to a publishing error, Figure 3 (parts A and B) in the above paper were reproduced incorrectly. The correct complete Figure 3 is shown below.

## Figures and Tables

**Figure 3:**
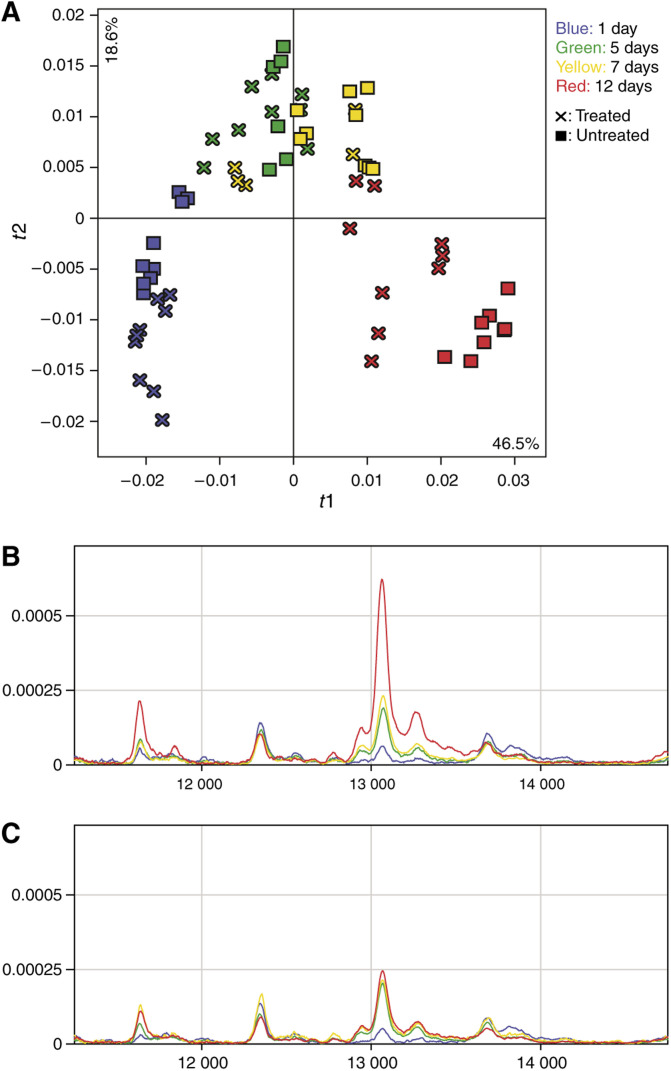
Principal component analysis reveals a time trend in protein expression during tumour progression (**A**). *R2X*(cum)=0.65 for the first two components. × and ▪ represent treated and untreated samples, respectively, and the different colours represent samples collected at different time points after treatment, as follows: blue=1 day; green=5 days; yellow=7 days; red=12 days. (**B**) and (**C**) display mean protein profiles within a specific spectral region, derived from untreated and treated samples, respectively. The samples are from the same time points as above and the same colour coding is applied.

